# Study Protocol of Brief Daily Body-Mind-Spirit Practice for Sustainable Emotional Capacity and Work Engagement for Community Mental Health Workers: A Multi-Site Randomized Controlled Trial

**DOI:** 10.3389/fpsyg.2020.01482

**Published:** 2020-06-26

**Authors:** S. M. Ng, Herman H. M. Lo, Albert Yeung, Daniel Young, Melody H. Y. Fung, Amenda M. Wang

**Affiliations:** ^1^Department of Social Work and Social Administration, The University of Hong Kong, Pokfulam, Hong Kong; ^2^Department of Applied Social Sciences, The Hong Kong Polytechnic University, Kowloon, Hong Kong; ^3^Department of Psychiatry, Harvard Medical School, Boston, MA, United States; ^4^Department of Social Work, Hong Kong Baptist University, Kowloon, Hong Kong

**Keywords:** work engagement, burnout, body-mind-spirit (BMS) practice, community mental health workers, randomized controlled trial (RCT)

## Abstract

**Background:**

Given the emotional demanding nature of social services, we developed a brief daily body-mind-spirit (BMS) program and successfully piloted it with workers at elderly services. The proposed study focuses on community mental health workers who are often under chronic stress and vulnerable to burnout.

**Methods:**

The study aims to evaluate the program for fostering sustainable emotional capacity and work engagement for community mental health workers. A multi-site randomized controlled trial design is adopted. All the 24 the Integrated Community Centre for Mental Wellness (ICCMW of Hong Kong will be approached to join this program. Assuming conservatively, 60% ICCWM (14 centers) will respond and participate. At each site, a pair of intervention and control groups will be run. The targeted total sample size is 224. To investigate the course of changes in burnout and engagement, each group will last 6 months, including 3-month intervention and 3-month follow-up. Measures will be taken at monthly intervals.

**Discussion:**

In light of literature and the pilot trial’s findings, participants in the Brief Daily BMS intervention group are expected to have a reduced burnout level and a narrowing of range in work engagement during the 3 months intervention. And within the 3 months post-intervention period, a rebound of burnout level and a widening of range in work engagement are expected to be observed in the same group of participants. Hopefully, this study will contribute to the deeper understanding of burnout and work engagement, and shed light on sustainable intervention for emotionally demanding workplaces.

**Clinical Trial Registration:**

The trial has been registered in the Clinical Trials Centre of the University of Hong Kong. HKUCTR-2763 Registered 27 December 2019 – Retrospectively registered, http://www.hkuctr.com/Study/Show/eb930d24e2c647afb7a922055163f24b.

## Background

### Community Mental Health Workers in Need of Continued Emotional Support

Work burnout is identified with three elements: cynicism, exhaustion and reduced efficacy (Maslach et al., 2001). Burnout is common in all sectors - governmental, commercial and non-governmental organizations. In positions where job demands/resources matching is strained, employees often experience chronic stress and are vulnerable to burnout (Schaufeli and Bakker, 2004). Prolonged intense work stress is associated with health and mental health problems (Sanderson and Andrews, 2006). At an organizational level, productivity and quality suffer (Lerner et al., 2004). There are more sick leaves, work injuries and staff turnover. The continued trend of globalization and advance in information technology has intensified competition and threatened job security in many industries. The relentless demands for productivity and quality enhancement are both real strains and psychological stresses for workers.

The welfare sector is no exception and staff well-being at workplace is a concern. Two common characteristics of human services work, intensive emotional involvement and humanitarian values, would function as vulnerable factors. Social workers who experienced the overwhelming emotional involvement, as a form of emotional labor, will be exposed to emotional exhaustion, which is a key characteristic of work burnout (Maslach et al., 2001). Also, it is common to have discrepancies between one’s personal/professional values and the reality of work, which may induce intense psychological conflicts. For some workers, a coping mechanism would be a disengagement from work (Park and Folkman, 1997). Moreover, an individuals’ apathetic attitude can affect co-workers as well as the morale of a work team (Schaufeli and Bakker, 2004).

In the context of Hong Kong, the extreme work-life imbalance is common and adds to the stress of workers (Ng et al., 2009b, p. 179). In many work teams, overtime work is a norm rather than the exception, and members are under pressure to conform at the cost of sacrificing some personal and family time. In the welfare sector, additional stresses include relentless pressure on securing funding, fulfilling elaborative documentation procedures, meeting the increasing expectations of service users, and in some cases, dealing with unfriendly resistance from local residents. There is a widespread feeling among social service workers in Hong Kong that job burnout has become an epidemic (Ng, 2008). A worrying indicator is persistently high staff turnover, with double-digit annual staff turnover rate being a norm.

The proposed study focuses on the well-being of community mental health workers. In 2010 the Social Welfare Department, Hong Kong SAR government restructured the fragmentized community mental health services around a new mega, one-stop service named ICCMW (Ng, 2015, p. 8). The scope of service of ICCMW is extremely wide, ranging from education and prevention to treatment and rehabilitation. Targeted service users include people suffering from SMI and common mental disorders, as well as healthy people in the community. Operational modes include rapid response to psychiatric emergency, time-limited casework, long-term support for people with chronic SMI, day center and community development. As a result of the service restructure, a total of 24 ICCMWs were established covering the whole territory of Hong Kong, which has a population of over 7 million. The 24 ICCMWs are manned by a totally of about 700 mental health workers. Most of them have a background in social work, nursing and occupational therapy. Charged with such a wide scope of responsibility, ICCMWs are overstretched to achieve all the goals and pledged service outputs (Ng, 2015, p. 36). Moreover, due to the nature of work, community mental health workers often spend much time in performing home visit on their own. This makes it difficult to create a supportive teamwork environment. As a result of all these factors, community mental health workers are under chronic stress and are vulnerable to burnout. They need continued support for sustainable emotional capacity and work engagement.

### A Paradigm Shift: From Symptoms-Reduction to Fostering Positive Well-Being

In social services, human capital is the most important asset of the organization. It is critically important to sustain and foster the staff’s well-being. Regarding interventions, there are practical limits concerning what may be done at the organizational level due to the operational constraints, such as contracts with the funding bodies and expectations of service users. Nevertheless, previous studies have suggested that while the tangible incentives are meaningful, they are not the paramount factors to influence the engagement and work burnout (Schaufeli and Bakker, 2004).

At individual levels, existing studies mainly concentrate on the symptoms reduction and have only shown quite limited short-term effects (Schaufeli and Bakker, 2001; Richardson and Rothstein, 2008; Goldgruber and Ahrens, 2010). Moreover, a symptoms-reduction mode would over-simplify work stress as negative, which is conceptually wrong and unhelpful. Some workers may see the ‘symptoms’ as evidence for requesting more tangible benefits from the employers. The purpose of instilling ownership of one’s personal well-being is defeated.

Our studies in Hong Kong have demonstrated the effects of holistic care culture in alleviating burnout and promoting work engagement (Ng et al., 2011; Fong et al., 2016). The updated researches about well-being in the workplace tend to treat work engagement as an outcome measure. In opposition to burnout, work engagement characterized as three components: passion, vigor, and absorption at work of employees (Schaufeli and Bakker, 2004; Fong and Ng, 2012; Ng, 2014). While appearing to be opposite concepts, work engagement and burnout have been revealed to be rather independent, demonstrating negative correlation at only mild to moderate magnitude (Schaufeli and Bakker, 2004; Gonzalez-Roma et al., 2006; Ng et al., 2011). Otherwise speaking, lack of burnout and existence of engagement may not necessarily happen at the same time. Because burnout and engagement influence well-being independently (Hakanen and Schaufeli, 2012), both of them need to be addressed.

A new tendency is adopting a positive-oriented approach to enhance workplace well-being (Gonzalez-Roma et al., 2006; Ng et al., 2011; Fong et al., 2016). One of these positive approaches is to foster daily spiritual experience, which showed encouraging possibilities (Holland and Neimeyer, 2005). Daily spiritual experience can be understood as the interaction between a person’s daily behavior/emotion and the transcendence in ordinary life (Underwood, 2006; Ng et al., 2009a). For instance, feeling blessed by a higher power, finding peace and strength deep inside, and experiencing intimate transpersonal connections. Previous studies show that a sound correlation existed between daily spiritual experience and well-being (Ellison and Fan, 2008).

Because of the tight association among BMS, holistic care should include all the three elements in fostering well-being (Ng and Chan, 2005, p. 71). In practice, the usual starting point is to link with the body (Ng, 2009, p. 128). Building the connection with their own body increases people’s awareness and helps to ground the self in the present moment. Being more grounded, people may broaden their awareness and build a connection with the mind and spirit. Brief BMS practices have been evaluated and assessed with plenty of evidences in a wide range of clients, for instance, people with mood and sleep disturbances, chronic fatigue, and survivors of critical incidents (Ng et al., 2006; Chan et al., 2016, 2017; Ji et al., 2017). Given the emotional demanding nature of social services, we developed a brief daily BMS program and successfully piloted it with both professional and care workers at elderly service (Ng, 2014). The proposed study aims to further and more rigorously evaluate the efficacy of the program.

### What We Have Done: Piloted a Feasible Brief Daily Body-Mind-Spirit (BMS) Program and Validated Scales Essential for Pursuing Rigorous Trials

#### The Brief Daily Body-Mind-Spirit Program for Social Service Workers

It is challenging to develop a brief daily BMS program that is feasible for busy social service workers of varied cultural and educational backgrounds. The program should pay attention and respect to religious pluralism, including not only one single religion, as well as spiritual, but non-religious orientation or practice. Finally yet importantly, this intervention should be concise otherwise it would become a burden for the practitioners, including facilitators.

Taking into consideration the above mentioned, we cooperated with a major elderly community center in Hong Kong and applied a brief daily wellness program providing 15 min BMS practice for their staff in a format of small group on every workday. The targeted group size is around 8–12. We trained a social worker of this center as the group’s facilitator. The group sessions are delivered in a compact and quiet room within their center. Sitting on the floor with a yoga mat created a relaxing environment for the group members. For those who are difficult to sit on the ground, chairs were provided. Each session runs about 15 min and will contain three standardized sections: Cooling down, Proverb sharing and Energy activity. The contents of the intervention will be described in the method section of this manuscript.

We have piloted this brief daily BMS program separately for professional and care workers at elderly services for a duration of 1 month (Ng, 2014). Both qualitative and quantitative findings supported the acceptability and feasibility of the intervention, and its potential efficacy in reducing burnout and fostering sustainable emotional capacity and work engagement. Attendance rate in both groups exceeded 80%. More encouragingly, the dropout rate was zero in both groups. In subsequent focus group meetings, participants shared that they felt revitalized with positive energy after every meeting, and thus could better work with service users at workplace and enjoy the time with family members in the evening. Both the facilitators and participants enjoyed participating in the program and did not see it as an additional burden. Some participants expressed that they actually looked forward to it on every workday during the pilot period, and wished that the program could be continuously offered at their workplace.

The quantitative findings of the pilot trials are also encouraging. Despite a small sample size (totally *N* = 16), reduction in burnout was significant in both groups. Effect size was in the range of moderate magnitude. However, the positive changes at 1 month after completion of the program appeared to diminish. This suggests that a continued or a longer duration program is desirable.

Findings of change in work engagement go beyond the expectation, but perhaps are most inspiring. In the group with a high baseline engagement level (4.0 out of a theoretical range of 0–6; *SD* = 0.8), a significant reduction in engagement was observed, in parallel to a mark reduction in burnout. In the group with an average baseline engagement level (3.0; *SD* = 1.0), no significant change in engagement level was observed, but in parallel, there was also a mark reduction in burnout.

A tenable theory is that a higher work engagement level is not always desirable. Several studies indicate the “dark side” of “over-engagement”: extremely high level of work engagement can be counterproductive to individual well-being and organizational performance (Bakker et al., 2011). For example, over-engagement may lead to workaholics, unbalance between work and life, and harm to interpersonal relationships (Beckers et al., 2004; Beal et al., 2005). Furthermore, it is also unrealistic to expect that employees are kept “engaged” all the time. Bakker et al. (2011) argues that perhaps a fluctuating level of work engagement is desirable.

The finding of our trial and previous literature shed the light on the understanding of work engagement: an optimal range of engagement level may exist. A good analogy is that in an endurance race, the one who presses him/herself too hard will sooner or later drop out of the race. Perhaps engagement is like a ‘fire’ in our heart fueling us at the workplace. However, if the ‘fire’ is overly strong, it will ‘burn’ us. The findings from our pilot trials seem to suggest that the appropriate intervention goal with work engagement is to facilitate participants finding an optimal, sustainable level. Neither too low nor too high is desirable.

#### Chinese Utrecht Work Engagement Scale (C-UWES)

The original Utrecht Work Engagement Scale (UWES) has 17 items covering 3 domains, namely Dedication, Absorption, and Vigor (Schaufeli et al., 2002). We validated this scale with 992 workers in more than 30 Hong Kong elderly service centers. After careful exploratory and confirmatory factor analysis, we removed the unsatisfactory items and produced a 9-item solution consisting of the same 3 domains of Vigor, Dedication and Absorption (Fong and Ng, 2012). Cronbach’s alpha coefficients of this shortened scale were 0.88 for the whole scale, and 0.70 to 0.77 for the 3 subscales.

#### Chinese Daily Spiritual Experience Scale (C-DSES)

Daily spiritual experience (DSE) refers to a person’s perception of involvement of the transcendence in daily life (Underwood and Teresi, 2002). After qualitative study and repeated exploratory and confirmatory factor analysis on different populations, Underwood and Teresi (2002) developed the Daily Spiritual Experience Scale (DSES) which is a single factor, 16-item self-report scale. The items measure experience rather than particular beliefs or rituals, and intend to transcend religious and cultural boundaries. The scale has high internal consistency with Cronbach’s alpha at 0.95, as well as good construct validity. DSES has attracted researchers from different countries, including France, Spain, South Korea, and Vietnam (Underwood, 2006). Ng et al. (2009a) validated the Chinese DSES in Hong Kong Chinese population and revealed robust psychometric properties of the 16-item single factor structure.

#### Body-Mind-Spirit Well-Being Inventory (BMSWBI)

In response to the growing popularity of multidimensional BMS interventions and a corresponding need for a multidimensional outcome measure, Ng et al. (2005) developed BMSWBI, a self-report multi-item scale with 4 factors, namely Physical Distress, Positive and Negative Affect, Daily Functioning, and Spirituality. The scales have high reliability, with alpha coefficients ranging from 0.87 to 0.92. The tool has been used in a wide range of health studies, and the scale validation paper has been cited over 90 times in academic publications (Google Scholar, 2016).

#### Research Gap and Significance of the Proposed Study

At a practical level, community mental health workers of ICCMW are working under chronic stress and are vulnerable to burnout. However, they lack sufficient and continued emotional support. In light of the promising findings in the pilot trials, the brief daily BMS program may be an effective and feasible intervention for these social workers in mental health service to foster their emotional capacity and work engagement. In this regard, a more rigorous trial of the program is worth pursuing. If the eventual research results are positive, they know how of fostering sustainable emotional capacity and work engagement will be transferable to other emotionally demanding workplaces.

At a theoretical level, the study will reveal a new understanding of work engagement. Our pilot trials revealed a reduction of exceptional high engagement alongside with reduction in burnout. And previous studies also suggest the level of work engagement is not the higher the better and some negative consequences may be caused by over-engagement (Beckers et al., 2004; Beal et al., 2005; Bakker et al., 2011). Engagement may be like ‘fire’ fueling us at work, but it can ‘burn’ if it is overly strong. While most researches on work engagement focus on its positive consequences, studies on the “dark side” is inadequate (Crawford et al., 2010; Christian et al., 2011; Bailey et al., 2017). Therefore, this study will enrich the understanding of work engagement and is expected to find an optimal range for work engagement.

## Research Methods and Design

### Research Aim and Objectives

The overall aim of the proposed research is to pursue the further rigorous trial of the brief daily BMS program, which demonstrated good potentials in the pilot studies. The specific objectives are:

(1) To evaluate the efficacy of the brief daily BMS program in reducing burnout and enhancing engagement for community mental health workers;
(2) To examine the trajectory of burnout, especially exhaustion, during a 3-month intervention and 3-month post-intervention periods; and
(3) To examine the trajectory of engagement during a 3-month intervention and 3-month post-intervention periods.

### Study Design

A multi-site RCT design is adopted. All the 24 ICCMWs will be invited to join this research. Assuming a conservative response rate of 60–70%, about 14–17 ICCMWs will join. At each site, a pair of intervention (brief daily BMS group) and control groups (tea break group) will be run. Each group will have about 10 participants. Thus, there will be a totally about 280–340 participants randomly assigned to either an intervention or control group. The study will be registered with the Clinical Trials Centre of The University of Hong Kong.

### Hypotheses

In light of the literature and findings of the pilot trials, we have the following hypotheses:

H1: Reduction of burnout will be observed within the 3-month intervention period;
H2: Rebound of burnout will be observed within the 3-month post-intervention period;
H3: A narrowing of range in work engagement will be observed within the 3-month intervention period; and
H4: A widening of range in work engagement will be observed within the 3-month post-intervention period.

### Study Setting

This study will use a two-armed randomized-controlled trial design to compare the intervention group (BMS group) and control group (Tea break group). Researchers will promote the program in all 24 ICCMWs in Hong Kong and eligible participants will be recruited from the ICCMWs.

### Eligibility Criteria

This study welcomes both professionals (such as social worker, occupational therapist, and nurse) and supporting staff (such as peer support worker and administration). However, to ensure the attendance rate, only full-time staff are included while freelance or part-time workers will not be recruited in this study.

#### Intervention

Based on the successful experience of the pilot study, each session of the brief daily BMS intervention will run about 15 min on each working day and contain three standardized sections:

(1) Cooling down (about 4 min): It usually functions as the first step in spiritual or religious practices. The main purpose is to remind practitioners to bring their awareness to the present moment. In this way, they may build a connection with their body, mind and the environment they present at the moment (Ng et al., 2017). Examples to serve this objective include sitting on the floor or chair silently, counting the breath in and out. It is also helpful to play soft music or natural sounds, like birds sing or sounds of waves. The facilitator may spontaneously pick one of these practices in each group session.
(2) Proverb sharing (about 8 min): Almost every culture has many proverbs, also known as mantras or sayings, using a simple sentence to provide life advice and philosophy. The group leader will prepare one positive sentence related to meanings of life, in order to provoke reflection and discussion within the group. Some examples are “*When slowing down, we can see the direction of cloud*,” and “*Compared with advance, compromise usually needs more courage and wise.*”
  The facilitator will deliver the sentence to each member in paper or through a cell phone. In the beginning, all participants, including the facilitator read this sentence out together. And then all members reflect it in silence for about 1 min. Thereafter, they will share their view about the sentence freely in the group. The aim of this activity is to stimulate thinking about purpose and meaning of life.
(3) Energy activity (about 3 min): This activity serves as an ending ritual for the group with the purpose of provoking positive energy, enabling members to revitalize after the group, and fostering a mutual care atmosphere among the members. Some samples can be physical exercise, stretching, singing an inspirational song or interaction movement, such like mutual massage or hugging game. Regarding the physical contact, cultural sensitiveness should be borne in mind. The group leader may choose one of these exercises freely to end up a group session.

Because the activities in the three sections are standardized and fairly straightforward, the facilitators will be chosen from group members and given some prior training. Specially developed group activities toolkits will be provided to the facilitators.

### Participants and Sample Size Calculation

In light of the findings of the pilot trials, an effect size is assumed at a moderate level of 0.4. For a 2-arm RCT with power at 90%, significance level at 0.05, and allowing for a 10% attrition rate (dropout rate in pilot studies was zero), a sample size of 75 per arm is needed (G^∗^Power software version 3.1, Franz Faul, University of Kiel, Germany). Totally 150 participants will be required. Assuming an average of around 8 participants per group, a total of 9 pairs of intervention and control groups will be needed. Each study site, an ICCMW, will host a pair of intervention and control groups. Thus, a totally 9 study sites are needed.

The other consideration is the variability among the 24 ICCMWs in Hong Kong. They are run by 11 NGOs with a rather diversified background. For example, some of these NGOs are more experienced in mental health services, and some are bigger and more well-established. To reduce the bias at the study-site level, instead of only randomly selecting 9 of them, we aim to invite all the 24 ICCMWs to participate in the study. Assuming a conservative response rate of 60%, about 14 ICCMWs will join. At each site, a pair of intervention and control groups will be run. Each group will have about 8 participants. Thus, there will be a totally about 224 participants randomly assigned to either an intervention or a control group.

### Recruitment Strategy

There are a totally 24 ICCMWs in Hong Kong. Each ICCMW has around 30 mental health workers. Both PI and co-I’s are experienced mental health social work researcher/practitioners and have a long-term collaborative relationship with the NGOs providing mental health services in Hong Kong. The PI is also the Consultant of the Mental Health Social Work Chapter of the Hong Kong Social Workers Association. The Chapter, with most members from ICCMWs, is concerned about the chronic stress and lack of emotional support experienced by community mental health workers. With a neutral, academic affiliation, the PI and co-I’s are in a good position to invite ICCMWs to participate in the study.

By means of computer-generated random numbers, ICCMWs will be invited to participate in the study one by one. The PI and co-I’s will initiate meeting and site visit with each ICCMW according to the sequence generated by computer. If an ICCMW agrees to join the study, the PI and co-I’s will hold a briefing session for all the mental health workers of the Center and invite them to participate in the study. Those showing interest and providing informed written consent will be randomly assigned to either an intervention or control group at a 1:1 ratio with the aid of computer-generated random numbers. The recruitment process will continue with all the 24 ICCMWs.

### Procedures

In order to examine the course of changes in burnout and engagement, the intervention period is extended from 1 month in pilot trials to 3 months in the proposed study. The post-intervention follow-up period is also extended to 3 months. Measures will be taken at baseline (T0), and then at monthly intervals (T1–T6). All questionnaire surveys will be administered by the research assistant of the study.

According to the experience of pilot trials, frontline professionals such as social workers, occupational therapists and nurses are good facilitators for the intervention groups. A 1-h training session suffices in introducing new facilitators to the intervention. A member of the research team will sit-in the meetings of each new group in the first week, and provide feedback to the facilitator. Afterward, to ensure treatment fidelity, a member of the research team will visit the group biweekly, until the end of intervention.

During the 3-month intervention period, the trained facilitator will run the brief daily BMS group in a quiet room at the site every workday, normally 5 times a week except when there are public holidays. At each site, the facilitator and participants may decide on their preferred time slot on a day for the BMS group that fits their work schedule. It can be in the morning, at noon or in the afternoon, and the exact meeting time can be changed during the 3-month intervention period if the group prefers. The facilitator will keep an attendance record of participants for statistical purpose.

**FIGURE 1:**
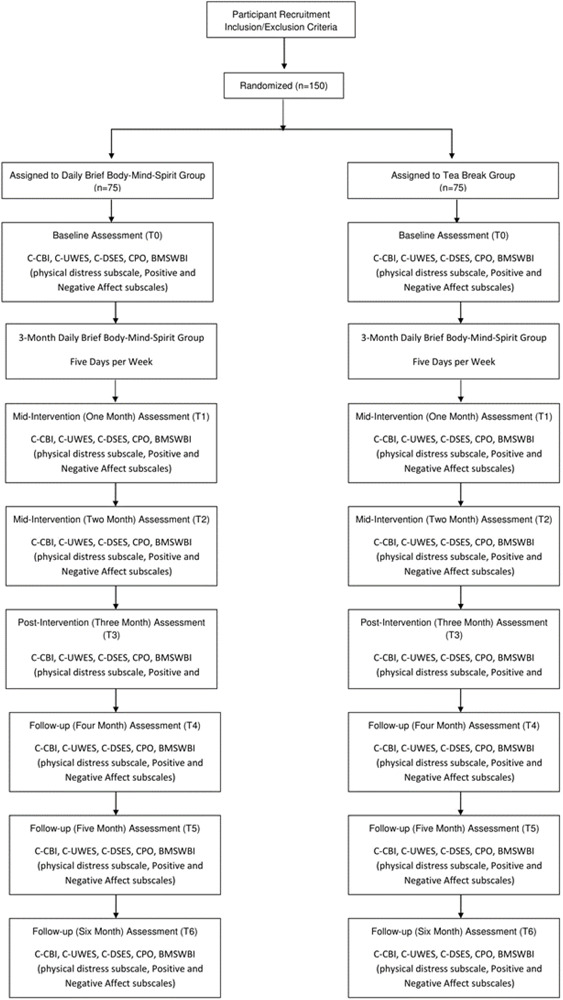
CONSORT diagram of intervention and active control groups and data collection points.

To control for the social gathering effects of the experimental group, a control group in the format of daily 15-min tea break will be organized in parallel at the same site on every workday during the 3-month intervention period. A participant of the control group will be assigned as the coordinator who will take care of the basic logistics, such as room and materials booking, time keeping, and keeping an attendance record of participants. The tea-break is to be conducted in a quiet room provided with the usual drinks and snacks available for staff at the site. The tea-break is unstructured. During the 15 min, participants are free to enjoy the drinks and snacks, and interact with other group members. Similar to the intervention groups, each control group may decide on their preferred time slot on a day for the tea-break that fits their work schedule. The same set of questionnaires will be administered on participants of each control group by the research assistant of the project at baseline (T0), then at monthly intervals (T1–T6). Figure 1 CONSORT diagram depicts the flow of the trial.

### Outcomes Measures

Measures of the related variables are summarized in Table 1 below. In line with the study’s objectives, primary outcome variables are work burnout and engagement. Since the intervention adopts a multidimensional BMS orientation, multiple somatic complaints, positive and negative affect, and daily spiritual experience are included as secondary outcome variables. Also included as secondary outcome variables are worker behavior, including overtime hours, sick leave, and injuries at work.

**TABLE 1:** Variable table.

**Variable/instrument**	**Content of the instrument**	**Data type**
**Primary outcome variables**		
**(1) Demographics** ◆ Self-report items	Age, gender, marital status, family composition, education and professional qualification, religious belief	Ordinal and Nominal
**(2) Work burnout** ◆ Copenhagen Burnout Inventory-Chinese Version (Fong et al., 2014)	19-item self-report scale with 3 sub-scales: personal burnout, work-related burnout, and client-related burnout	Interval
**(3) Work engagement** ◆ C-UWES (Fong and Ng, 2012)	9-item self-report scale with 3 factors: Vigor, Dedication, and Absorption	Interval
**Secondary outcome variables**		
**(1) Multiple somatic symptoms** ◆ Physical Distress subscale of the Body-Mind-Spirit Well-Being Inventory (Ng et al., 2005)	14-item self-report scale	Interval
**(2) Positive affect** ◆ Positive Affect subscale of the Body-Mind-Spirit Well-Being Inventory (Ng et al., 2005)	8-item self-report scale	Interval
**(3) Negative affect** ◆ Negative Affect subscale of the Body-Mind-Spirit Well-Being Inventory (Ng et al., 2005)	11-item self-report scale	Interval
**(4) Daily spiritual experience** ◆ Chinese Daily Spiritual Experience Scale (Ng et al., 2009a)	16-item self-report scale	Interval
**(5) Collective psychological ownership** ◆ Chinese Collective psychological ownership Scale (Ng and Su, 2018)	7-item self-report scale	Interval
**(6) Worker behavior** ◆ Self-report items	Overtime hours, sick days taken off from work and injuries at work in the past month	Interval and ordinal

### Data Analysis

Both completers and intention-to-treat analysis will be performed. In the intention-to-treat analysis, missing data will be handled by ‘last observation carried forward.’ With a 2-arm RCT design, the efficacy of the intervention will be examined by repeated measures MANOVA on both primary and secondary outcome variables. Partial eta-squared (η^2^) values are computed to assess the effect sizes; values of 0.02, 0.13, and 0.26 suggest small, medium, and large effect sizes, respectively (Pierce et al., 2004). To investigate within group effects, serial trend analysis from T0 to T6 will be conducted on the outcome variables. In the case of work engagement, changes in a range of scores from T0 to T6 will also be examined. The associations between primary and secondary outcome variables will be explored by correlational and multiple regression analyses.

### Tentative Schedule

Duration of the proposed study is 30 months. Since totally 14 pairs of intervention and control groups will be required, a longer period of intervention is required. Detailed planning of research activities is depicted in the project chart in the next section.

## Discussion

As symptom reduction approach has only demonstrated limited effects in the previous studies, positive outcomes and overall wellbeing needed to be included as the purpose of a workplace intervention (Ramaci et al., 2019). Fostering spiritual experience in daily life, as one of the positive-approach interventions showed encouraging potentials (Holland and Neimeyer, 2005). Daily spiritual experience can be understood as the interaction between a person’s daily behavior/emotion and the transcendence in ordinary life (Ng et al., 2009a). Because of the robust association among BMS, holistic care should include all the three elements in fostering well-being (Ng and Chan, 2005, p. 71). Brief BMS practices have been built up and assessed with evidences in a wide range of clients, including people with mood and sleep disturbances, depression, chronic fatigue, and survivors of critical incidents. The proposed study has several practical and theoretical implications.

### Practical Implications

At the practical level, compared with the other staff wellbeing or mindfulness/self-awareness interventions, this proposed study will demonstrate that a continued or long duration well-being program can be feasible, effective and sustaining for the community mental health workers, who have to frequently cope with high emotional demanding situations. Most staff well-being programs are one-off in design, and can hardly produce sustaining effects. On the other hand, the popular Mindfulness-based Stress Reduction (MBSR) program is fairly demanding, requiring 2.5-h weekly session and 45-min daily homework exercise for eight consecutive weeks (Janssen et al., 2018). The acceptability of MBSR program is limited among the busy health care professionals. More brief mindfulness intervention has drawn the attention of researchers (Zeller and Levin, 2013; Strauss et al., 2018).

The proposed Brief Daily BMS group only takes 15 min for a daily session, which can be conveniently led by one of the group members. The flexible meeting schedule may integrate the practice into the members’ daily routine – making the program more accessible and sustainable for those busy professionals. Furthermore, the proposed intervention combined self-awareness/mindfulness components with other activities, including golden sentence sharing and physical exercise. It may contribute to the call for diverse development of mindfulness interventions and positive well-being approach in the workplace (Barattucci et al., 2019). The proposed protocol may serve as a reference model for developing effective, practical program addressing the needs of mental health workers in diversified settings. Moreover they know how of fostering sustainable emotional capacity and work engagement may be transferable to other emotionally demanding workplaces.

### Theoretical Implication

At the theoretical level, this study will contribute to answering the debate whether intervention with self-awareness/mindfulness component can reduce burnout significantly. Self- awareness/mindfulness interventions demonstrated a promising effect to reduce work related stress (Strauss et al., 2018). However, the results of these interventions on burnout are equivocal. Significant improvement of burnout has not been observed in some previous studies (Cohen-Katz et al., 2005; Janssen et al., 2018). This may be partly because the widely used measure, Maslach Burnout Inventory (Maslach et al., 1996) cannot capture the core components of burnout (Lomas et al., 2017). The proposed study will adopt the Copenhagen Burnout Inventory – Chinese Version, which has been revealed to show satisfactory validity and reliability (Fong et al., 2014). The results of our study will contribute to the evaluation of burnout relief from self-awareness/mindfulness interventions.

Besides, the study will reveal a new understanding of work engagement. Our pilot trials revealed reduction of exceptional high engagement alongside with reduction in burnout. Previous studies mentioned above also show that if the engagement level is “too high,” it may undermine individual wellbeing as well as organizational performance. Therefore, engagement may be like ‘fire’ fueling us at work, but it can ‘burn’ if it is overly strong. This study is expected to reveal an optimal range for work engagement: high enough for good performance, low enough to avoid burnout. Furthermore, because engagement consists of three components: passion, vigor, and absorption, the multidimensional analysis may shed light on the effect of each component of work engagement.

## Conclusion

Work related stress and burnout among employees have become a concern in virtually all sectors. This is especially relevant to community mental health workers who are often under intensive emotional labor. After several decades of research and trials, there is a general trend of moving toward a more holistic and positive-oriented approach in workplace wellbeing intervention. In view of the emotional demanding nature of social services, we developed a brief daily BMS program which has been successfully piloted with both professional and care workers at elderly services (Ng, 2014). The proposed study aims to further more rigorously evaluate the efficacy of the program with community mental health workers.

With active control (tea break group), this brief daily BMS intervention will offer 15-min session (with three standardized components) for the staff in small group on each working day. This study will measure both burnout and work engagement, as well as examine the trajectory of both constructs among the participants during a 3-month intervention and 3-month post-intervention periods.

In light of literature and the pilot trials’ findings, participants in the brief daily BMS intervention group are expected to have a reduced burnout level and a narrowing of range in work engagement during the 3-month intervention period. And within the 3-month post-intervention period, a rebound of burnout level and a widening of range in work engagement are expected to be observed among the participants. Hopefully, this study will contribute to the deeper understanding of burnout and work engagement, as well as shed light on sustainable intervention for emotionally demanding workplaces.

## Data Availability Statement

The datasets generated for this study are available on request to the corresponding author.

## Ethics Statement

The studies involving human participants were reviewed and approved by the Human Research Ethics Committee of The University of Hong Kong. The patients/participants provided their written informed consent to participate in this study.

## Author Contributions

SN designed the study, liaised with the study sites, and monitored implementation of the study. HL, AY, and DY advised on the design and implementation of the study. MF and AW helped to implement the study and collect the data. All authors contributed to the article and approved the submitted version.

## Conflict of Interest

The authors declare that the research was conducted in the absence of any commercial or financial relationships that could be construed as a potential conflict of interest.

## Abbreviations

BMS: Body-Mind-Spirit
BMSWBI: Body-Mind-Spirit Well-being Inventory
CBI: Copenhagen Burnout Inventory
C-DSES: Chinese Daily Spiritual Experience Scale
Co-I: co-investigator
CPO: Chinese Collective Psychological Ownership Scale
C-UWES: Chinese Utrecht Work Engagement Scale
ICCMW: the Integrated Community Centre for Mental Wellness
MANOVA: multivariate analysis of variance
PI: principal investigator
RCT: randomized controlled trial
SMI: severe mental illness.
